# Functional and sensory properties of toasted tortillas are shaped by structural changes in native maize starch

**DOI:** 10.3389/fnut.2025.1695996

**Published:** 2025-12-08

**Authors:** Gabriela Palacios-Pola, Hugo R. Perales, Juan de Dios Figueroa Cárdenas, Miguel Abud-Archila, Anayansi Escalante-Aburto, Lurline Álvarez Rateike, Susana Del Carmen Bolom Martínez, Verónica Lagunes Quevedo

**Affiliations:** 1 Faculty of Nutrition and Food Sciences, Universidad Autónoma de Ciencias y Artes de Chiapas, Tuxtla Gutiérrez, Mexico; 2 El Colegio de la Frontera Sur, San Cristobal de las Casas, Mexico; 3 Centro de Investigación y de Estudios Avanzados Unidad Querétaro, Santiago de Querétaro, Mexico; 4 Tecnológico Nacional de México/Instituto Tecnológico de Tuxtla Gutiérrez, Tuxtla Gutiérrez, Mexico; 5 Institute for Obesity Research, School of Engineering and Sciences, Tecnologico de Monterrey, Monterrey, Mexico

**Keywords:** tostadas, nixtamalization, resistant starch, sensory attributes, structural properties

## Abstract

The majority of maize-based foods are produced via nixtamalization, which includes toasted tortillas (tostadas). Women artisanal producers in Chiapas, Mexico, have refined key quality parameters, such as texture and resistant starch content, by using native maize varieties that preserve traditional traits. Nixtamalization modifies maize functionality through the formation of resistant starch, which resists digestion and supports beneficial colonic microbiota via short-chain fatty acid production. This study aimed to evaluate the functional, sensory, and structural properties of tostadas that were prepared via two cooking processes using native maize from Chiapas. In this research, starch gelatinization was achieved through three different processes: traditional nixtamalization (control), short boiling, and full boiling. The resulting tostadas were: nixtamalized corn tostadas (NCT), partially-burst tostadas (PBT), and fully-burst tostadas (FBT), respectively. Tostada sensory attributes were analyzed by a trained panel that assessed multiple parameters, namely crunchiness, fracturability, hardness, corn aroma, nixtamal aroma, corn taste, nixtamal taste, and stickiness. On the other hand, consumer testing was used to evaluate chewing parameters. Partially-burst tostada (PBT) and fully-burst tostada (FBT) exhibited structural, functional, and sensory properties that are associated with the sensation of satiety and liking. Corn races did not have a statistically significant effect on the rheological or sensory properties of tostadas. However, the nixtamalization method significantly influenced stickiness and taste parameters. Stickiness intensity (4.50) and eating rate (62.66 g/min) for FBT were statistically different from PBT’s respective values (3.75 and 56.12 g/min). These characteristics favor the chewing process. When maize is cooked for longer periods, the peak of amylose–lipid complexes is more easily detected. Resistant starch in FBT was higher (5.6%) than in PBT and NCT, which were 3.7% and 2.4%, respectively. Key tostada quality parameters, such as chewiness and sensory characteristics, correlated well with the structural and rheological properties of the modified starch (partial gelatinization) formed during tostada preparation.

## Graphical abstract

**Figure:**
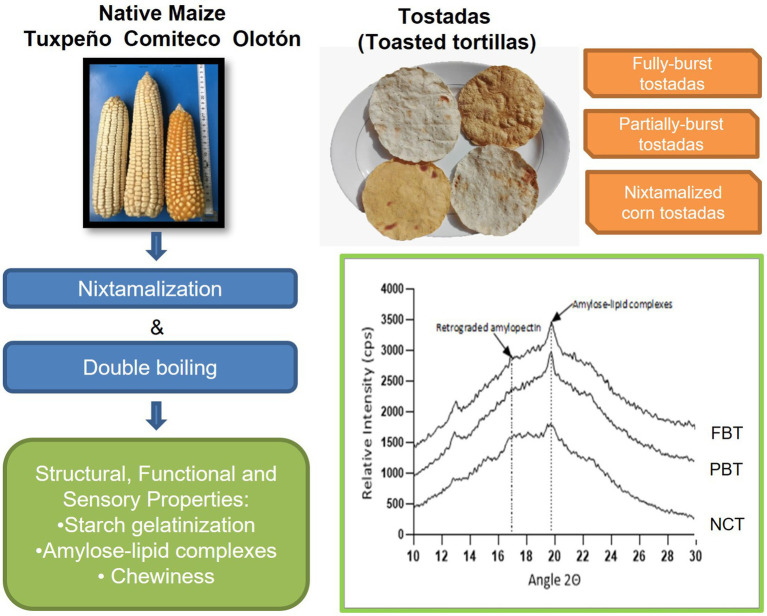

## Introduction

Maize (*Zea mays* L.) is the most widely consumed cereal in Mexico (1). Preparation of maize-based products requires the kernels to undergo a thermal treatment in an alkaline medium, a process known as nixtamalization. This process enhances the nutritional profile of maize by increasing the bioavailability of niacin, improving protein quality, and reducing mycotoxin content. Nixtamalization yields a dough (masa) that serves as the foundational ingredient for a wide range of maize-based foods, both at industrial and domestic levels. These foods, produced using the nixtamalization process, include tortillas, tostadas, tortilla chips, tamales, and pozole (2). The nixtamalization process involves three major steps. The first step is cooking the maize in a suspension of lime (calcium hydroxide) for approximately 30 min. Then, the nixtamal (cooked maize) is allowed to rest in the cooking fluid, called nejayote, for 12 h. Afterward, the nixtamal is rinsed twice with water and ground into *masa* dough before finally being used to prepare various maize products. In this study, a nixtamal double-cooking process is introduced, consisting of cooking maize in a lime solution for 30 min, after which the nixtamal is washed to remove the nejayote. The clean nixtamal is then boiled again in water for an extended period. The second cooking phase ends when the previously nixtamalized kernels burst. Finally, the kernels are left to rest for 16 h in an alkaline or water solution (3, 4).

In Chiapas, Mexico, the industrial nixtamalization follows a standardized process; however, G. Palacios (personal communication, 23 August 2022) noted that many women continue to carry out this procedure using artisanal methods that vary significantly across regions. Such diversity in traditional practices results in considerable variability in the quality of maize-based products. While tortillas continue to be the primary product derived from maize, tostadas—in their various forms, including chips, totopos, and baked tostadas—represent a growing and economically relevant market (5). As with tortillas, the quality of tostadas is also influenced by the specific nixtamalization process employed. Qualitative studies (6) have shown that even within the same community, and among neighboring households, tortillas and tostadas may exhibit distinct sensory properties and physicochemical characteristics. Moreover, women who make and sell artisanal maize tostadas in the Altos of Chiapas region, Mexico, have made efforts to achieve adequate textures that increase chewing ease (4).

Tostadas have two important quality attributes, namely a lower breaking force and a higher crunchability (7). Tostadas should have a high breaking force to resist the functionality while using it as a spoon or for dipping, or as a temporary plate to hold food on it. At the same time, it should also break easily to allow easy chewing during mastication (8). To achieve that quality effect, women use one or two cooking periods after rinsing the nixtamal (cooked maize). However, the second boiling is done without lime to achieve a grain puffing, which augments “fluffiness,” where the grain expands its volume, and other compounds derived from gelatinization are formed. These are known as resistant starch type 5, which are amylose–lipid complexes with functional properties (9). Some functional foods have properties that are influenced by starch, which contributes toward control of viscosity, moisture, consistency, mouthfeel, texture, and shelf life (10).

Biochemical reactions and molecular interactions occur in both cooking phases and during resting periods. These steps affect the structure of amylose and amylopectin (11), causing different degrees of gelatinization in the outer and inner layers of the endosperm (12). The amylose–lipid complexes that occur due to interactions between the hydrophobic fraction of amylose and lipids have been described as a new source of resistant starch (RS5). Their presence results in a reduction of postprandial glycemic and insulin responses and potential benefits for intestinal health (13). Figueroa et al. (14) indicated that Mesoamerican societies, such as the Maya and Aztec, used annealing and heat-moisture treatments of starch granules in the preparation of traditional dishes such as tamales, pozole, and atoles. These high-processing temperatures cause amylose–lipid Type I complexes to turn into a higher-perfection Type II crystal and increase the gelatinization temperature of the starch granule. In turn, this produces a resistant starch (RS) consisting of amylose–lipid inclusions (RS5). Our hypothesis is that this double-cooking process of nixtamal creates tostadas with an improved texture (crisp and soft), with a high content of resistant starch, involving less chewing effort for the consumer. There are no previous reports linking maize cooking and its impact on the properties of tostadas. Therefore, this study aimed to evaluate the functional, sensory, and structural properties of tostadas that were prepared via two cooking processes with native maize from Chiapas.

## Materials and methods

### Materials

Corn samples from Olotón (O), Comiteco (C), and Tuxpeño (T) races, grown in areas near San Cristóbal de las Casas and Zinacantán (O), Comitán, Las Rosas, and Venustiano Carranza (C), and Chicoasén and Amatenango del Valle (T), were used in this study. Tostadas were prepared by female artisanal producers from the communities of Carrizalito (San Cristóbal de la Casas), Yalumá (Comitán), and Teopisca (Teopisca), using corn from each of the races mentioned above.

### Maize cooking

**Figure 1:**
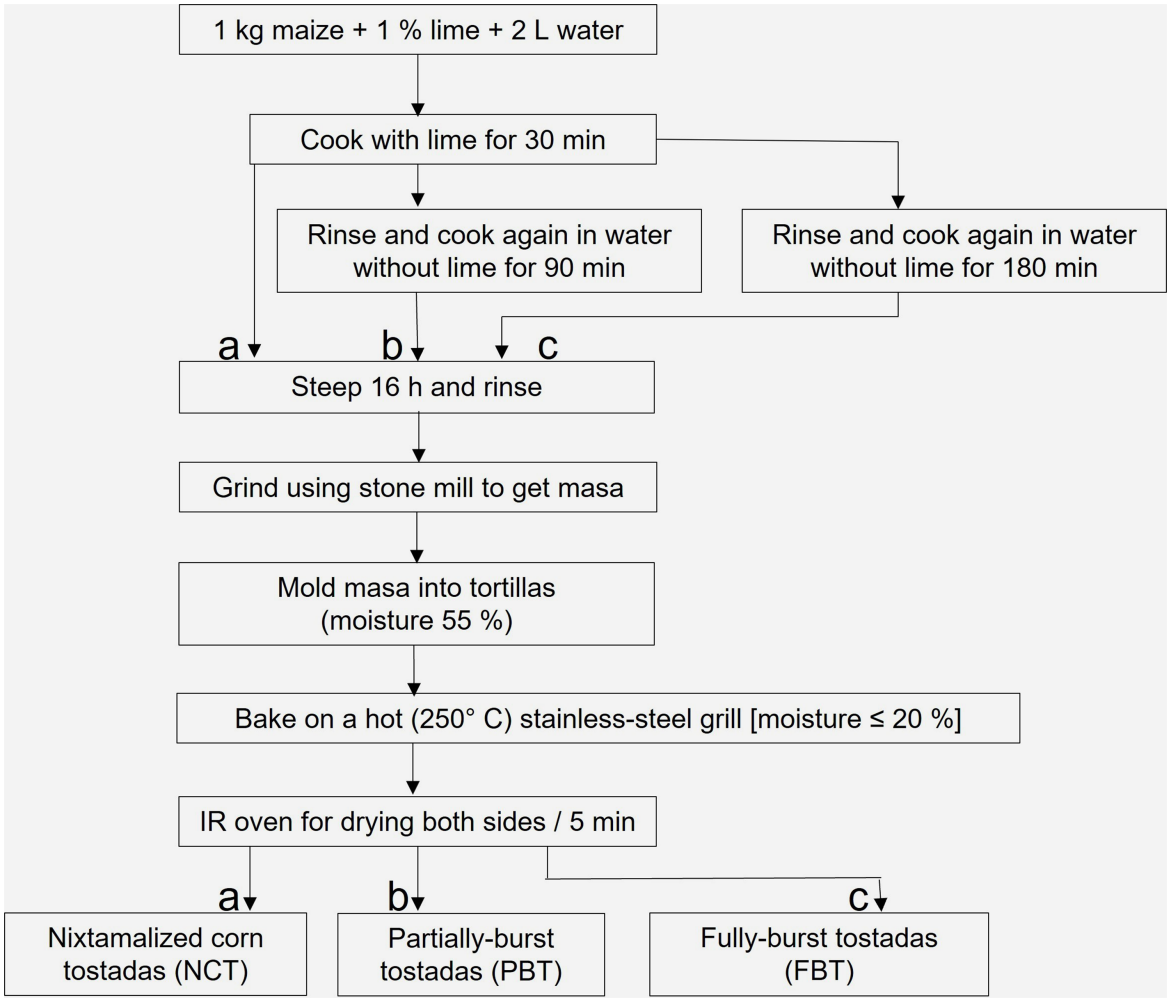
Flow diagram of the process for elaborating NCT, PBT, and FBT.

The tostadas were prepared using dough obtained from each maize race, which was nixtamalized separately. Three treatments were used for each race: The first cooking phase for all three treatments (nixtamalization) consisted of cooking the maize for 30 min in an alkaline solution (1% lime). The first treatment allows the nixtamal to rest for 16 h and does not undergo a second cooking, while the other two do. In both cases, once the nixtamal has cooled, the alkaline residual water (nejayote) is drained, and clean water is added for the second cooking: a short 90-min cooking and a long 180-min cooking. The nixtamal is then left to rest for 16 h. In all three treatments, after resting, the nixtamal is rinsed and ground in a wet mill. When the dough reaches a moisture content of 55%, thin discs of the dough are formed using a manual tortilla press. The tortillas were cooked on griddles (250 °C) until the moisture content was reduced to 20%. Finally, both sides were heated with infrared heat for 5 min, transforming into toasted tortillas (Figure 1). Toasted tortillas from the first treatment were coded as NCT, and those from the other two treatments were coded as PBT and FBT, depending on the processing time of the second cooking: PBT for the short second cooking and FBT for the long second cooking.

### Sample preparation

All tostadas were maintained at a constant moisture of 7% and analyzed in triplicate. The tostada samples were pulverized and sieved through a US No. 60 sieve for thermal, rheological, chemical, and X-ray diffraction analyses. Another set of tostada samples was kept in their original shape for the evaluation of physical and sensory properties.

### Tostada properties evaluation

#### Physical properties

Thickness and diameter were measured with a digital caliper (model CD-6, Mitutoyo, Kanagawa, Japan). Moisture was determined by oven-drying, according to method no. 945.15 (15). Luminosity was determined with a Hunter Lab Colorimeter (MiniScan^®^ XE model, Reston, VA, USA) (16). Hardness, fracturability, and crunchability were determined with a TA-XT2 texturometer (Texture Technologies Corporation, Stable Micro Systems, Godalming, UK) equipped with a 19.03 mm diameter spherical TA-18A probe and a TA-94 extrusion platform. The texture profile was carried out at a test speed of 5 mm/s. The breaking strength of the samples was determined by placing the probe in the center of each piece and compressing until breaking of the sample. The maximum force was recorded as hardness in Newton units.

#### Chemical properties

Resistant starch and total dietary fiber concentration were determined using test kits (Megazyme, Bray, Ireland) based on approved methods AACC 32-40.01 and AACC 32-05.01, respectively (17). Moisture (925.09), crude protein (954.00), fat (920.39 and 963.15), ash (923.03), and crude fiber (985.29) were analyzed according to AOAC (18). Food energy content was calculated according to a method reported by FAO (19).

#### Thermal and rheological properties

Thermal properties were measured using a Differential Scanning Calorimeter (DSC Q200, TA Instruments, DE 19720, United States) following the methodology described by Santiago-Ramos et al. (12). Rheological properties were measured with a Rapid Visco-Analyzer (RVA-model 3C, New-Port Scientific, Sydney, Australia), according to Narváez-González et al. (20).

Finally, a Rigaku DMAX-2100 (Rigaku Corp, Tokyo, Japan) was used for analyzing pulverized tostada samples by X-ray diffraction. Operating conditions were 30 kV and 16 mA, CuKα radiation was *λ* = 1.5405, and the measurements were carried out from 5° to 50°, 2Ɵ, as reported by Mariscal-Moreno et al. (21).

### Sensory analysis

Sensory analyses were carried out with a trained panel (*n* = 8). The study was approved by our institutional ethics committee (approval number FCNA-CIBBOO1-2021). The QDA methodology (22) was performed, measuring eight attributes (crunchy sound, fracturability, hardness, corn aroma, nixtamal aroma, corn taste, nixtamal taste, and stickiness) in tostada samples. For consumer testing (*n* = 126), overall acceptance levels were measured using a nine-point hedonic scale (23). Chewing time, the number of chewing and swallowing required (for one portion), was also measured (24), as well as satiety levels (high, medium, and low), adapted from Bennett and Blissett (25). The eating rate was calculated from the amount of tostada in grams and the chewing time in minutes (26). The consumer study with tostadas was conducted with a total of 126 untrained panelists, of whom 40% were men and 60% women, aged between 18 and 25 years. The surveys were administered with the participants’ informed consent, and all panelists were notified that they could withdraw from the evaluation at any time.

### Statistical analysis

The results of all evaluated properties were analyzed using ANOVA tests with Minitab software (Minitab Inc., 2010). Data are presented as means and standard deviations, using Tukey’s test was applied for pairwise comparison of means. Differences among samples were considered statistically significant at *p*-value ≤ 0.05.

## Results and discussion

### Physical and chemical properties of tostadas

When maize was exposed to the cooking process for a longer time (FBT), a higher impact on the physical properties associated with the texture was found (Table 1). The tostadas with the lowest fracturability level (6.5 N) were those made with the FBT process. Additionally, crunchiness levels did not differ statistically between the NCT and FBT processes. Moreover, the fracturability (ranging between 7.5 and 8 N) and crunchability (ranging between 13.5 and 15.7 N) of tostadas showed no statistical differences (*p* > 0.05) between corn races. As for toasted tortilla hardness, levels resulted in a statistically significant difference between FBT (7.5 N) and PBT (10.5 N). These values exceed those reported in the literature for plant-based nutritional snacks, which typically range between 2.3 and 3.4 N (27). FBT shows lower hardness and fracturability but high crunchability, and resistant starch represents better textural and nutraceutical quality than the NCT and PBT treatments. The resistant starch values were 5.6% for FBT, 3.7% for PBT, and 2.4% for NCT (Table 1). The levels of resistant starch for FBT and PBT were significantly higher than the values reported by Campus-Baypoli et al. (28) and by Santiago-Ramos et al. (29) for tortillas, where values are 2.5% and 3.1%, respectively. These RS values are important because the resistant starch type 5 (RS5) is the portion of starch that is not absorbed or decomposed in the human small intestine, which is partially or totally fermented in the large intestine with the formation of short-chain fatty acids. RS5 causes direct effects on consumer health, as it prevents colon cancer and maintains low levels of cholesterol and glycemic index in blood (30). Dietary fiber was not significantly different between NCT, PBT, and FBT. This may be due to reactions generated by continuous exposure to heat, such as pyrodextrinization of starch and the production of free polysaccharides, which increase indigestible carbohydrates (31).

**Table 1:** Physical and chemical properties of tostadas.

Treatment	Thickness	Weight	Diameter	Luminosity	Hardness	Fracturability	Crunchability
	(mm)	(g)	(mm)	(L*)	(N)	(N)	(N)
NCT	ND	ND	ND	65.9 ± 1.1 a	9.8 ± 3.6 a	8.5 ± 4.2 a	18.4 ± 4.5 a
PBT	1.9 ± 0.3 a	14.8 ± 3.3 b	14.9 ± 2.2 a	64.6 ± 2.1 a	10.5 ± 4.7 a	9.0 ± 4.9 a	13.4 ± 4.1 b
FBT	1.8 ± 0.3 a	17.7 ± 4.7 a	12.5 ± 1.7 b	65.3 ± 2.1 a	7.5 ± 2.6 b	6.5 ± 2.4 b	16.1 ± 4.2 a
Olotón	1.9 ± 0.3 a	15.4 ± 3.9 a	14.3 ± 3.1 a	64.8 ± 1.9 ab	8.3 ± 2.4 a	7.5 ± 2.4 a	15.1 ± 3.7 a
Comiteco	1.9 ± 0.3 a	17.6 ± 3.4 a	12.6 ± 1.6 a	63.9 ± 1.9 b	9.3 ± 4.5 a	8.0 ± 4.5 a	15.7 ± 4.9 a
Tuxpeño	1.8 ± 0.3 a	16.1 ± 5.1 a	14.2 ± 1.7 a	66.2 ± 1.9 a	8.9 ± 5.0 a	7.8 ± 4.9 a	13.5 ± 4.4 a

Treatment	Moisture	Ash	Protein	Crude Fiber	Fat	Carbohydrate	Energy	Resistant starch	Dietary fiber
	(%)	(%)	(%)	(%)	(%)	(%)	(kcal/100 g)	(%)	(%)
NCT	9.2 ± 0.1 b	1.5 ± 0.2 a	9.8 ± 0.4 a	1.0 ± 0.3 b	4.1 ± 0.3 a	74.0 ± 0.2 a	371.6 ± 3.1 b	2.4 ± 0.9 b	6.5 ± 0.5 a
PBT	9.4 ± 0.4 ab	1.3 ± 0.1 a	9.4 ± 0.4 a	1.2 ± 0.3 b	4.3 ± 0.2 a	74.6 ± 0.8 a	374.6 ± 0.6 a	3.7 ± 1.7 b	7.7 ± 0.4 a
FBT	9.7 ± 0.1 a	1.4 ± 0.1 a	8.9 ± 0.9 a	1.8 ± 0.3 a	4.0 ± 0.4 a	74.6 ± 1.6 a	369.6 ± 0.8 b	5.6 ± 1.6 a	7.8 ± 1.0 a

The values (mean ± standard deviation) followed by the same letter in the same column within the same group are not significantly different (*p* ≤ 0.05). NCT, nixtamalized corn tostadas; PBT, partially burst tostada; FBT, fully burst tostada. ND, Not determined.

### Rheological and thermal properties of tostadas

Table 2 shows the thermal and rheological properties of NCT, PBT, and FBT tostadas made with three native maize types from Chiapas. For PBT, retrogradation (SBV) and final viscosity (FV) values were higher (693 cP and 1935 cP) than for FBT (469 cP and 1,654 cP), as extending the heat treatment drives reactions between lipids and amylose, decreasing the viscosity of gelatinization and slowing down retrogradation (32). Mariscal-Moreno et al. (21) reported that the formation of type-5 amylose complexes decreases retrogradation due to the interaction of the two phenomena: competition with the retrogradation process by the amylose present in starch, and the second, by the formation of complexes between lipids and amylopectin. Moreover, maize races did not statistically influence the thermal and rheological properties of tostadas.

**Table 2:** Effect of the nixtamalization process on rheological and thermal properties of tostadas.

					Endotherm 1 (retrogradation)			
Treatment	PT (°C)	PV (cP)	SBV (cP)	FV (cP)	To (°C)	Tp (°C)	Tf (°C)	–ΔH (J/g)
NCT	NF	NF	NF	NF	40.9 ± 4.1 a	43.4 ± 4.3 a	49.7 ± 3.9 a	0.82 ± 0.60 b
PBT	90.9 ± 0.0 a	1,313 ± 238 a	693 ± 310 a	1,935 ± 457 a	38.3 ± 3.5 a	45.1 ± 4.7 a	51.4 ± 6.1 a	1.97 ± 1.41 a
FBT	90.9 ± 0.0 a	1,203 ± 260 a	469 ± 127 b	1,654 ± 339 b	39.5 ± 4.1 a	44.9 ± 4.9 a	52.2 ± 5.2 a	1.48 ± 0.76 ab

	Endotherm 2 (gelatinization)				Endotherm 3 (RS5)			
Treatment	To (°C)	Tp (°C)	Tf (°C)	ΔH (J/g)	To (°C)	Tp (°C)	Tf (°C)	–ΔH (J/g)
NCT	64.0 ± 5.7 a	66.3 ± 6.8 a	76.9 ± 6.9 a	0.43 ± 0.23 a	NF	NF	NF	NF
PBT	63.0 ± 9.0 a	67.6 ± 11.2 a	77.00 ± 13.1 a	0.76 ± 0.98 a	100.6 ± 9.7 a	107.2 ± 8.2 a	114.3 ± 9.1 a	0.53 ± 0.62 a
FBT	60.4 ± 8.5 a	66.5 ± 5.6 a	75.8 ± 8.1 a	0.77 ± 0.70 a	94.2 ± 10.5 a	99.5 ± 11.6 a	107.6 ± 9.6 a	1.58 ± 1.64 a

The values (means ± standard deviation) followed by the same letter in the same column are not significantly different (p ≤ 0.05). PT, pasting temperature; PV, peak viscosity; SBV, setback viscosity; FV, final viscosity; To, onset temperature; Tp, peak temperature; Tf, final temperature; –ΔH, enthalpy; NCT, nixtamalized corn tostadas; PBT, partially-burst tostada; FBT, fully-burst tostada; NF, not found.

The enthalpy shown during retrogradation for PBT was higher (1.97 J/g) than that for FBT (1.48 J/g), although the enthalpy corresponding to gelatinization remained at similar values for PBT and FBT (0.76 and 0.77) but lower for NCT (0.43) (Table 2). The low gelatinization enthalpy is explained by NCT, derived from single alkaline cooking, thus showing no greater increase in gelatinization caused by double cooking. Additionally, overcooked nixtamal loses its ability to retain water, resulting in a dough with reduced moisture content. The excessive breakdown of structural components renders the dough soft, weak, and unable to form a cohesive structure due to the increased degree of starch hydrolysis (33).

In NCT, the characteristic endotherm of RS5 formation was not observed (Table 2). RS5 enthalpy is higher in FBT (1.58 J/g) compared with the value of 0.53 J/g found for PBT. The PBT and FBT starches presented an enthalpy change (ΔH) higher than that found in chickpea and bean nixtamalized flour (0.59 and 0.39) (34, 35). This enthalpy is associated with the amylose–lipid complexes, which reduce the retrogradation of starch for delaying the recrystallization of amylopectin (21).

### X-ray diffraction patterns

**Figure 2:**
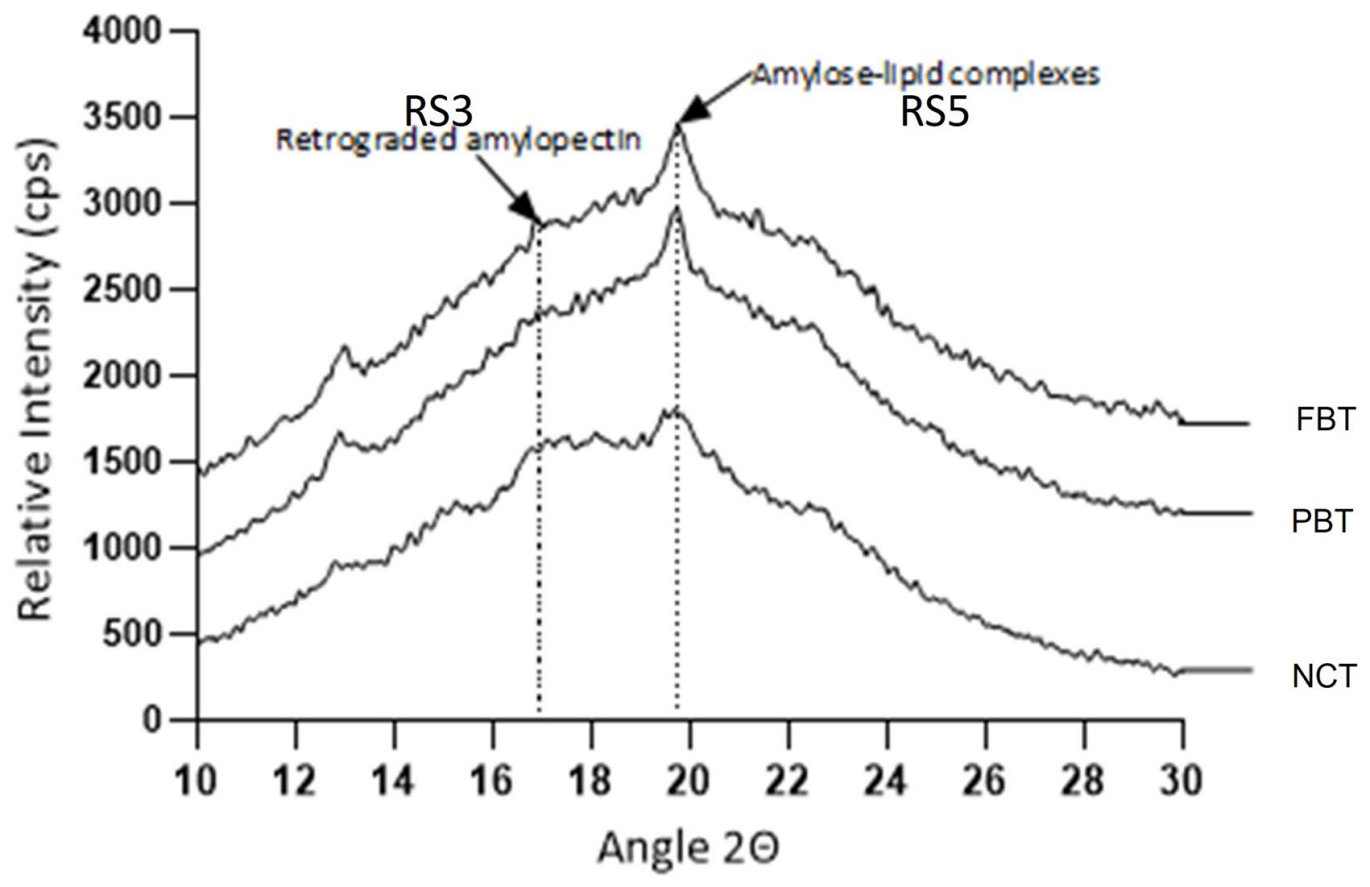
Peaks from X-ray diffractometry indicated RS3 and RS5 in NCT, PBT, and FBT.

The X-ray diffractogram shows the peaks formed in NCT, PBT, and FBT, as can be seen in Figure 2. At approximately 17° angle 2Ɵ, a retrograded starch is indicated, corresponding to RS3 (enzyme-resistant starch). This peak is best appreciated in NCT. Amylose is mainly responsible for retrogradation because its linear chains are joined through hydrogen bonds, integrating them into a network that begins to grow or thicken as storage time passes; so the higher the content of amylose, the greater the retrogradation (36).

After gelatinization of granular starch, amylose and amylopectin recrystallize into molecules with type-5 structures, corresponding to RS5 (amylose–lipid complexes), with pronounced peaks close to 20° angle 2Ɵ in PBT and FBT. Processing conditions, especially extended cooking time/heating, influence resistant starch formation in tostadas in both PBT and FBT, especially in the longer cooking time (FBT) treatment (Table 2).

### Sensory properties of tostadas

The superior sensory characteristics of Olotón and Comiteco native maize tostadas were associated with texture, flavor, and overall taste (Table 3). Nixtamal taste and stickiness (with values of 3.78 and 4.5, respectively) were perceived with higher intensity in the FBT samples, as compared to 3.17 and 3.75 in PBT samples (Table 3). However, the maize race did not show any statistically significant effect on the evaluated parameters. Nixtamal flavor is perceived by the chemical reactions occurring between calcium hydroxide and the amino acids of proteins (37), while stickiness is a textural attribute linked to the food’s viscosity that is generated by the formation of amylose–lipid complexes, creating a network that provides less resistance to biting. Chewiness, as a textural property, describes the energy required to chew food until it is ready for consumption (27). The chewing process required less time and fewer chews in FBT samples (34.64 s and 37.31 chews) compared to PBT samples (38.85 s and 40.85 chews). However, no significant differences were detected in deglution number, and the eating rate was higher in the FBT samples samples (Table 3). Satiety showed no differences among treatments for the evaluated maize races. The scale provided contained three levels, and all variables studied showed an intermediate level of satiety. It may be useful to explore this parameter by expanding the scale to determine if there are consumer subgroups with different satiety patterns compared to the total sample of consumers.

**Table 3:** Sensory properties of tostadas determined by (A) panelists and (B) consumers.

(A)	Corn flavor	Nixtamal flavor	Corn taste	Nixtamal taste	Crunchy Sound	Fracturability	Hardness to break	Stickiness
Effect of nixtamalization process								
PBT	3.45 ± 1.61 a	3.10 ± 1.72 a	4.69 ± 1.93 a	3.17 ± 1.46 b	4.67 ± 2.08 a	6.02 ± 1.28 a	3.44 ± 1.59 a	3.75 ± 1.98 b
FBT	3.81 ± 1.85 a	3.20 ± 1.87 a	5.08 ± 2.03 a	3.78 ± 1.77 a	4.54 ± 1.97 a	5.83 ± 1.46 a	3.27 ± 1.37 a	4.50 ± 2.15 a
Effect of race								
Olotón	3.59 ± 1.66 a	3.08 ± 1.61 a	4.81 ± 1.95 a	3.35 ± 1.54 a	4.24 ± 1.94 a	5.85 ± 1.38 a	3.41 ± 1.44 a	3.78 ± 2.13 a
Comiteco	3.59 ± 1.71 a	3.04 ± 1.89 a	4.88 ± 2.12 a	3.50 ± 1.72 a	4.79 ± 2.14 a	5.83 ± 1.36 a	3.68 ± 1.67 a	4.47 ± 1.93 a
Tuxpeño	4.06 ± 1.76 a	3.34 ± 1.89 a	4.94 ± 1.91 a	3.57 ± 1.68 a	4.81 ± 1.96 a	6.11 ± 1.38 a	2.94 ± 1.20 a	4.14 ± 2.21 a

(B)	Chewing time (s)	Chewing number	Deglutions number	Satiety level	Eating rate (g/min)	Texture	Flavor	Liking
Effect of nixtamalization process								
PBT	38.85 ± 16.19 a	40.85 ± 17.68 a	3.09 ± 1.34 a	2.22 ± 0.66 a	56.12 ± 28.12 b	5.10 ± 1.73 b	5.25 ± 1.64 b	5.40 ± 1.69 b
FBT	34.64 ± 15.13 b	37.31 ± 15.51 b	2.92 ± 1.36 a	2.21 ± 0.66 a	62.66 ± 29.31 a	7.27 ± 1.58 a	7.05 ± 1.71 a	7.36 ± 1.53 a
Effect of race								
Olotón	37.00 ± 15.95 a	38.26 ± 16.14 a	3.04 ± 1.30 a	2.21 ± 0.66 a	59.23 ± 29.64 a	6.15 ± 1.86 ab	6.11 ± 1.84 ab	6.43 ± 1.80 ab
Comiteco	37.81 ± 16.13 a	40.45 ± 17.23 a	3.02 ± 1.43 a	2.23 ± 0.66 a	58.79 ± 31.59 a	6.51 ± 1.86 a	6.50 ± 1.68 a	6.73 ± 1.64 a
Tuxpeño	35.47 ± 15.27 a	38.53 ± 17.71 a	2.96 ± 1.33 a	2.21 ± 0.65 a	60.10 ± 25.20 a	5.89 ± 2.31 b	5.83 ± 2.11 b	5.98 ± 2.13 b

The values (means ± standard deviation) followed by the same letter in the same column within the same group are not significantly different (*p* ≤ 0.05). PBT, partially-burst tostada; FBT, fully-burst tostada.

The attribute evaluated by the panelists with the best scores was the fracturability of tostadas (ease of breaking). The rheological properties of starch, as well as the mechanical and structural variations that are produced during chewing, are the variables that mediate the perception of the texture of semisolid foods (38). Furthermore, oral processing is dynamic and involves the specialized simultaneous detection of several sensors, which is why texture is considered a multiparametric sensory property (39).

The eating rate (Table 3) recorded from consumer data suggests that the values are comparable to those reported for various fruits and vegetables—such as peas, tomato purée, white rice, apple, smoked salmon, and meatballs—which are associated with low energy intake, as noted by Viskaal-van Dongen et al. (26). In FBT, the eating rate was 62.66 g/min and 56.12 g/min for PBT. Forde et al. (24) indicated values close to 60 g/min for boiled potatoes, raw tomatoes, and mashed carrots, while tortilla chips have an eating rate of 15 g/min. Tortilla chips induce a longer oral sensory exposure due to water–oil mass exchange, generating texture changes. Regarding energy content, the proximal chemical analyses performed on FBT (Table 1) showed lower energy intake (369.6 kcal/100 g) than commercial foods such as cheese (373 kcal/100 g) or rice waffles (380 kcal/100 g) (26).

These findings are very novel since some studies have reported that longer chewing times are important for reducing food intake (40). However, in this case, while chewing time is indeed important for the overall digestive process of starch, we would like to clarify that resistant starch type 5 (RS5) is an amylose–lipid complex. This complex is stabilized primarily by hydrophobic interactions and hydrogen bonding between the amylose helices and the lipid molecules. These molecular interactions prevent hydrolytic enzymes such as *α*-amylase and amyloglucosidase from effectively accessing and digesting the complex. As a result, the digestibility of RS5 is not significantly influenced by the eating rate. This structural resistance has been well-documented in the literature (41).

Similarly, the effects of higher dietary fiber content (RS5) are attributed to slowing the rate at which food leaves the stomach, which can extend the feeling of satiation after a meal. These lipid–starch complexes also reduce starch digestibility, further contributing to a slower release of energy and increased satiety (42).

However, in sensory terms, no link has been reported between RS5 content and oral food processing or sensory testing with consumers. Table 4 shows the Pearson correlation between RS5, the parameters of food oral processing, and sensory attributes. As shown, there are significant positive correlations between the number of chews and chewing time, which also exist between the level of liking and the sensory attributes of flavor and texture. This indicates that the greater the number of chews required, the longer the time invested in modifying the food before swallowing. Likewise, these findings confirm that consumer preference for tostadas is mainly driven by their flavor and texture. Another significant inverse correlation was observed between eating rate and chewing time and number, suggesting that higher values of these parameters are associated with slower feeding speeds. This finding is consistent with the results that were reported by Jan et al. (43) in their study regarding masticatory behavior. The correlation between texture, liking, and flavor with the chewing number was also inverse, albeit weak. This indicated that toasted tortillas were perceived as more pleasant when they required fewer chews. Conversely, texture and flavor showed a positive correlation with the eating rate. Finally, RS5 content did not show any significant correlation with sensory attributes or oral food processing parameters (*p* > 0.05).

**Table 4:** Pearson correlation between RS5 and parameters of food oral processing and sensory attributes.

	Chewing time	Chewing number	Deglutions number	Satiety level	Eating rate	RS5 content	Texture	Flavor	Liking
Chewing time	1								
Chewing number	**0.672** *	1							
Deglutions number	0.334 *	0.190 *	1						
Satiety level	0.235 *	0.205 *	0.150 *	1					
Eating rate	**−0.839** *	−0.631 *	−0.292 *	−0.156 *	1				
RS5 content	−0.564 0.056	−0.540 0.070	−0.491 0.105	−0.556 0.060	0.477 0.117	1			
Texture	−0.068 0.201	−0.122 *	−0.014 0.790	0.022 0.675	0.124 *	0.491 0.105	1		
Flavor	−0.124 0.019	−0.159 *	−0.038 0.473	−0.013 0.802	0.138 *	0.356 0.255	**0.821** *	1	
Liking	−0.074 0.162	−0.148 *	−0.029 0.582	−0.002 0.977	0.101 0.058	0.356 0.202	**0.863** *	**0.884** *	1

Values marked in bold close to 1 indicate a strong correlation. Positive values indicate a direct relationship, while negative values indicate an inverse relationship. Statistical significance was determined using a two-tailed test. *Correlation is significant at the *p* ≤ 0.05 level.

## Conclusion

The second cooking of nixtamal (FBT) positively affects tostada properties, especially structural, functional, and sensory features associated with satiety and preference. This process, which includes double cooking, grinding, baking, and toasting (FBT), causes important changes in starch through the formation of amylose–lipid complexes, which turn tostadas into functional foods due to their content of dietary fiber and resistant starch. In addition, the FBT process helps in obtaining food that is easy to store and is sought for its taste and texture (soft and crunchy) about as much as that of plant-based foods. Important parameters in food quality, such as chewiness and organoleptic characteristics, are related to the structural and rheological properties of altered starch during the preparation of tostadas. Tostadas obtained with PBT and FBT had characteristics expected from ultra-processed foods, such as their ease of consumption, low energy content, and eating rate, with potential effects contributing to the health and well-being of consumers due to their physiological effects. It is thus convenient to integrate them into the diet of overweight people or those with obesity problems.

Despite the findings reported, it remains important to pursue further research about the bioavailability of nutrients present in tostadas through both *in vivo* and *in vitro* assays. These approaches will allow for a more precise understanding of how these compounds are absorbed and utilized by the human body. Furthermore, it is important to establish correlations between the nutritional properties of tostadas and their potential impact on gut health, particularly in relation to the composition and activity of the intestinal microbiota. Such studies could provide valuable insights into the functional benefits of traditional maize-based products and contribute to the development of culturally relevant dietary strategies that promote overall health and well-being.

## Data Availability

The original contributions presented in the study are included in the article/supplementary material; further inquiries can be directed to the corresponding author.
